# Timing-Dependent Effects of Prebiotic–Probiotic Supplementation on High-Fat-Diet-Induced Testicular Dysfunction and Gut Microbiota Alterations in Rats

**DOI:** 10.3390/microorganisms14071566

**Published:** 2026-07-17

**Authors:** Ramtin Harati, Abdullah Burak Karaduman, Hulya Karaca, Busra Korkut-Celikates, Merve Guven, Merve Baysal, Gozde Aydogan-Kilic, Volkan Kilic, Ozlem Atli-Eklioglu, Ceren Ozkul, Sinem Ilgin

**Affiliations:** 1 Department of Pharmaceutical Toxicology, Faculty of Pharmacy, Anadolu University, Eskişehir 26470, Türkiye; ramtinharati@anadolu.edu.tr (R.H.); abkaraduman@anadolu.edu.tr (A.B.K.); busrakorkutcelikates@anadolu.edu.tr (B.K.-C.); mguven7@anadolu.edu.tr (M.G.); mbaysal@anadolu.edu.tr (M.B.); oatli@anadolu.edu.tr (O.A.-E.); 2 Department of Pharmaceutical Microbiology, Faculty of Pharmacy, Anadolu University, Eskişehir 26470, Türkiye; hulyakaraca@anadolu.edu.tr; 3 Department of Biology, Faculty of Science, Eskisehir Technical University, Eskişehir 26470, Türkiye; gozdea@eskisehir.edu.tr (G.A.-K.); vkilic1@eskisehir.edu.tr (V.K.); 4 Department of Pharmaceutical Microbiology, Faculty of Pharmacy, Hacettepe University, Ankara 06100, Türkiye; cerenozkul@hacettepe.edu.tr

**Keywords:** obesity, high-fat diet, male reproductive toxicity, prebiotic–probiotic supplementation, gut microbiome, inflammation, oxidative stress

## Abstract

Obesity is a chronic metabolic condition characterized by low-grade inflammation and oxidative imbalance and is strongly associated with impaired male reproductive function. This study investigated whether prebiotic–probiotic supplementation could prevent or attenuate high-fat diet (HFD)-induced metabolic inflammation-associated reproductive toxicity in male rats. Animals were assigned to four groups: normal diet (ND), HFD, HFD with concurrent prebiotic–probiotic supplementation from the onset of feeding (P-HFD), and HFD followed by supplementation initiated after 5 weeks (HFD-P). Evaluations included body and reproductive organ weights, sperm parameters, testicular histopathology, reproductive hormones, oxidative stress and inflammatory biomarkers, and gut microbiome composition. HFD feeding induced pronounced reproductive impairment, evidenced by reduced relative testicular weight, disrupted spermatogenesis, decreased LH levels, elevated TNF-α, a paradoxical increase in intratesticular testosterone despite reduced LH, and marked gut dysbiosis, characterized by shifts in microbial community structure along with reduced microbial diversity. Prebiotic–probiotic supplementation did not fully restore microbial richness or return the microbiota to an ND-like configuration; however, concurrent supplementation induced more pronounced taxonomic restructuring than delayed supplementation. While P-HFD did not show significant improvements in sperm parameters or LH, it exhibited clear histological preservation of testicular structure, reduced TNF-α, and partial normalization of testosterone, indicating attenuation of HFD-induced inflammation-associated testicular toxicity rather than complete functional recovery. In contrast, delayed supplementation produced no meaningful improvement in reproductive, hormonal, or microbiome-related outcomes and, in several respects, more pronounced testicular inflammation and structural degeneration than HFD alone. Collectively, these findings indicate that intervention timing is critical: concurrent administration conferred greater structural and anti-inflammatory protection against HFD-induced testicular damage, whereas delayed intervention was insufficient—or even counterproductive—once testicular injury was established. This timing-dependent response highlights the potential of microbiota-targeted strategies as supportive, timing-sensitive approaches for mitigating obesity-related male reproductive toxicity.

## 1. Introduction

Obesity is a chronic metabolic disorder resulting from an imbalance between energy intake and expenditure and has emerged as one of the most critical public health challenges worldwide [1]. According to the World Health Organization, approximately 43% of adults aged 18 years and older were overweight in 2022 [2]. Obesity is a significant risk factor for a wide range of metabolic and cardiovascular diseases [3,4,5]. Beyond its systemic consequences, accumulating evidence demonstrates that obesity also impairs male reproductive function. Excess body weight in men of reproductive age has been strongly associated with reduced fertility potential [4,6,7]. The mechanisms underlying obesity-induced male infertility are multifactorial, involving hormonal dysregulation, metabolic disturbances, and direct disruption of spermatogenesis. Among these mechanisms, chronic low-grade inflammation and oxidative stress are recognized as key mediators linking obesity to testicular dysfunction [7,8].

Given the multifaceted nature of obesity and its adverse reproductive consequences, there is an urgent need for safe, effective, and sustainable therapeutic strategies. Although lifestyle modification, pharmacotherapy, and bariatric surgery remain standard management approaches, their long-term effectiveness is often limited by poor adherence and adverse effects [9,10]. In recent years, modulation of the gut–testis axis via the gut microbiota has emerged as a promising complementary strategy. The gut microbiota plays a central role in regulating host metabolism, immune function, and systemic inflammation [1,11,12]. Dysbiosis, defined as an imbalance in microbial composition, has been implicated in obesity-associated metabolic and inflammatory dysfunctions [13,14,15]. Among available microbiota-targeted interventions, prebiotic or probiotic supplementation has attracted considerable attention. Restoring microbial homeostasis through prebiotic or probiotic supplementation has been proposed as a non-pharmacological approach for mitigating obesity-related complications [16,17,18,19].

Probiotics—defined as live microorganisms that confer health benefits when administered in adequate amounts—and prebiotics—non-digestible substrates that selectively stimulate the growth and activity of beneficial microbes—have both been shown to enhance gut barrier integrity, attenuate inflammation, and modulate host energy metabolism [20,21]. Clinical and experimental studies further demonstrate that prebiotics or probiotics interventions can reduce adiposity, improve insulin sensitivity, and alleviate obesity-associated metabolic disturbances [22,23]. Notably, multi-strain probiotic formulations containing high concentrations of *Lactobacillus*, *Bifidobacterium*, and *Streptococcus* species have exhibited pronounced anti-inflammatory, immunomodulatory, and metabolic effects, positioning them as promising candidates for targeting obesity-related dysbiosis [24,25]. Despite these advances, the underlying biological mechanisms by which modulation of the gut microbiota influences obesity-related male reproductive outcomes remain only partially understood. In particular, probiotic strains belonging to the genera *Lactobacillus* and *Bifidobacterium* have been widely reported to modulate gut barrier integrity, suppress endotoxemia, and attenuate low-grade systemic inflammation induced by high-fat diets [26,27,28]. Given that metabolic inflammation and gut dysbiosis are key upstream drivers of obesity-associated testicular dysfunction, multi-strain formulations incorporating these genera were considered biologically relevant candidates for investigating microbiota-targeted modulation of male reproductive outcomes. Accordingly, the selected prebiotic–probiotic formulation was designed to simultaneously target microbial composition, inflammatory tone, and metabolic signaling pathways implicated in obesity-related testicular injury.

Although several experimental studies have reported beneficial effects of probiotic or synbiotic supplementation on metabolic parameters and male reproductive outcomes, these studies exhibit important conceptual and methodological limitations that restrict their translational relevance. Most prior investigations have evaluated either preventive or therapeutic interventions in isolation, without directly comparing the impact of intervention timing within the same experimental framework. As a result, it remains unclear whether microbiota-targeted strategies can reverse established testicular injury or are effective primarily during the early stages of metabolic stress. Moreover, many previous studies have focused on a limited set of reproductive endpoints—such as sperm parameters or circulating hormone levels—without integrating parallel assessments of testicular histopathology, local inflammatory signaling, oxidative stress status, and gut microbiota composition. This fragmented approach has hindered a comprehensive understanding of how gut dysbiosis, inflammation, endocrine disruption, and structural testicular damage interact during obesity progression. Consequently, the timing-dependent reversibility of obesity-induced testicular dysfunction via modulation of the microbiota remains an unmet need in the current literature [29], limiting clinical translation of microbiota-targeted interventions for obesity-related male infertility.

Addressing these critical gaps, the present study directly compares concurrent versus delayed prebiotic–probiotic supplementation within the same high-fat-diet model. It integrates analyses of reproductive, endocrine, inflammatory, histopathological, and microbiome-level outcomes. By explicitly examining the role of intervention timing, this study aims to clarify whether microbiota-targeted strategies can prevent or reverse obesity-induced testicular dysfunction and to generate mechanistic insight into this timing-dependent response [30].

Accordingly, the present study investigated high-fat diet (HFD)-induced reproductive toxicity in male rats and aimed to characterize the biological correlates and candidate mechanistic pathways underlying this impairment. Comprehensive assessments included sperm parameters, testicular histopathology, and reproductive hormone levels (follicle-stimulating hormone (FSH), luteinizing hormone (LH), and testosterone), as well as oxidative stress markers (glutathione (GSH), malondialdehyde (MDA)), inflammatory mediators (tumor necrosis factor alpha (TNF-α), interleukin-6 (IL-6)), and gut microbiota composition. Given that HFD is known to induce marked alterations in gut microbial structure, HFD-associated microbial changes were further characterized using 16S rRNA gene sequencing. Furthermore, we evaluated whether prebiotic–probiotic supplementation, administered either concurrently with HFD feeding or following an initial HFD period, could mitigate HFD-induced disturbances in reproductive function, systemic biochemical and inflammatory profiles, and gut microbiota composition. By integrating microbiome, hormonal, biochemical, and histological analyses, this study aimed to determine the extent to which prebiotic–probiotic supplementation can alleviate HFD-induced reproductive impairments and to evaluate the influence of gut microbiota modulation on inflammatory and, secondarily, oxidative stress responses.

## 2. Material and Method

### 2.1. Experimental Animals and Study Design

One of the main challenges in establishing a reliable diet-induced obesity model in experimental animals is the lack of fully standardized protocols. Previous studies differ considerably in dietary composition, animal species, and feeding duration, complicating the development of a consistent, physiologically relevant model. In most established protocols, 45–60% of total caloric intake is derived from fat, and feeding periods of 3–4 weeks or more are required to initiate obesity. Although measurable weight gain typically becomes evident by the seventh week, extended feeding durations of 10–12 weeks are recommended to ensure full metabolic and phenotypic expression of obesity [31].

In this study, adult male Sprague Dawley rats (6–8 weeks old, weighing 180–200 g) were acclimatized for one week under standard laboratory conditions (24 ± 1 °C, 12 h light/dark cycle) with free access to chow and water. Animals were randomly assigned to four experimental groups (*n* = 10 per group; no formalized stratified or block randomization protocol was used) and housed in Plexiglas cages (three or four rats per cage), with different experimental groups housed in physically separate cages to prevent cross-group contact. All animals were individually coded, and samples for histological, sperm, and microbiome analyses were collected systematically from every animal without pre-selection; investigators were not blinded to group allocation during sample collection or analysis.

The high-fat diet consisted of 20% protein, 20% carbohydrate, and 60% fat, consistent with previously validated formulations [32,33,34]. The normal diet contained 34% protein, 50% carbohydrate, and 16% fat. Both diets were matched for micronutrient composition. The total feeding period lasted 10 weeks, allowing evaluation of HFD-induced reproductive alterations across approximately one complete spermatogenic cycle in rats, in accordance with OECD Test Guideline 416 [35]. In the concurrent supplementation group (P-HFD), prebiotic–probiotic administration was applied throughout the 10 weeks to assess its structural and anti-inflammatory protective effects during the entire spermatogenic cycle. In contrast, the 10 weeks were divided into two distinct phases in the delayed supplementation group (HFD-P) to specifically evaluate therapeutic effects. In this group, rats were exposed to HFD alone for the first five weeks to establish obesity-associated metabolic disturbances, followed by five weeks of continued HFD feeding combined with prebiotic–probiotic supplementation. This approach allowed direct comparison of preventive versus therapeutic intervention timing within a single spermatogenic cycle.

At present, there are no consensus-based guidelines for determining the optimal daily dose of combined prebiotic–probiotic formulations. Reported effective doses vary widely, ranging from 10^6^ to 10^11^ CFU/day [36]. The prebiotic–probiotic combination used in this study corresponded to a human-equivalent total daily dose of 1 × 10^10^ CFU/day of freeze-dried bacterial strains along with 300 mg/day of inulin. This dose was converted to the corresponding rat-equivalent dose using the body surface area-based Km conversion method [37], yielding an estimated dose of 1.03 × 10^9^ CFU/kg/day and 31 mg/kg/day of inulin. The formulation consisted of seven bacterial species, including four *Lactobacillus* species (*L. plantarum*, *L. acidophilus*, *L. rhamnosus*, *L. paracasei*) and three *Bifidobacterium* species (*B. lactis*, *B. longum*, *B. bifidum*). Although probiotic functionality is known to be strain-specific, the manufacturer’s product specifications did not include strain-level designations; therefore, strain-level identification could not be provided. The prebiotic component consisted exclusively of inulin, a non-digestible fructan widely used to selectively stimulate beneficial gut microbiota. It was administered concomitantly with the probiotic formulation throughout the designated supplementation periods.

Baseline body weights were recorded before the dietary intervention. Daily feed intake was regulated to approximately 10 g/day per rat; thus, cages housing four animals received 40 g/day, whereas cages containing three animals received 30 g/day. Actual daily feed consumption was calculated as the difference between the amount of feed provided and the remaining feed. Body weights were measured weekly at a consistent time of day, and animal health status and behavior (including general appearance, posture, and activity level) were monitored daily throughout the experimental period under a protocol approved by the institutional animal ethics committee (Anadolu University Local Ethics Committee for Animal Experiments, approval no. 2023-14). No signs of severe distress, morbidity, or adverse events were observed in any animal throughout the study.

The four experimental groups (*n* = 10 per group) were as follows:

- Normal Diet (ND) group: Rats were fed a standard control diet for 10 weeks.
- High-Fat Diet (HFD) group: Rats were fed a high-fat diet for 10 weeks.
- High-Fat Diet with concurrent Prebiotic–Probiotic supplementation (P-HFD) group: Rats were fed a high-fat diet and simultaneously administered a prebiotic–probiotic combination (1.03 × 10^9^ CFU/kg/day of freeze-dried bacterial strains and 31 mg/kg/day of inulin) via oral gavage for 10 weeks.
- High-Fat Diet followed by Prebiotic–Probiotic (HFD-P) group: Rats were fed a high-fat diet for the first five weeks, followed by an additional five weeks of high-fat feeding combined with daily administration of the prebiotic–probiotic combination at the same dose described above.

### 2.2. Sample Collection Procedures

#### 2.2.1. Blood Collection and Plasma Preparation

At the end of the 10-week experimental period, rats were humanely euthanized under intraperitoneal urethane anesthesia (1.5 g/kg; Sigma-Aldrich, Darmstadt, Germany). Blood samples were collected via cardiac puncture from the right ventricle as previously described [38,39,40,41]. Collected blood samples were maintained at 2–8 °C overnight, after which plasma was separated by centrifugation at 1000× *g* for 15 min. Plasma aliquots were stored at −20 °C until biochemical analysis.

#### 2.2.2. Testis and Epididymis Dissection

Following blood collection, the testes and epididymides were excised. The right testis was designated for histopathological evaluation, while the left testis and epididymis were rinsed with phosphate-buffered saline (PBS; MP Biomedicals, Illkirch, France), gently blotted dry, weighed, and homogenized for biochemical analyses. Tissue homogenates were divided into equal aliquots and stored at −20 °C for subsequent determination of GSH, MDA, TNF-α, IL-6, and testosterone levels using commercial assay kits.

For histological processing, the right testis was fixed in 10% neutral-buffered formalin (Sigma-Aldrich, Darmstadt, Germany) for 48 h, rinsed in running tap water, dehydrated through a graded ethanol series (Sigma-Aldrich, Darmstadt, Germany) (70%, 80%, 90%, 96%), cleared in xylene, and embedded in paraffin at 65 °C. Paraffin blocks were sectioned at 5 µm, floated in a 45 °C water bath, mounted on glass slides, and oven-dried for 1 h. Sections were subsequently deparaffinized in xylene, rehydrated through descending concentrations of ethanol, and stained with hematoxylin and eosin (H&E). After final dehydration and clearing, slides were mounted with Entellan and examined under an Olympus BH-2 light microscope (Olympus Optical Co., Ltd., Tokyo, Japan). Representative photomicrographs were captured using an Olympus DP-70 digital camera (Olympus Corporation, Tokyo, Japan) [38,39,40,41].

### 2.3. Sperm Parameters Analysis

The cauda region of the right epididymis was used to evaluate sperm parameters immediately after euthanasia. Sperm concentration, motility, and morphology were analyzed using a computer-assisted sperm analysis (CASA) system (Sperm Class Analyzer^®^ [SCA^®^], version 5.4.0.1; Microptic SL, Barcelona, Spain), coupled with a Nikon Eclipse 50i microscope (Nikon Instruments Inc., Tokyo, Japan; distributed by IMP, Johannesburg, South Africa), as previously described [38,39,40,41].

Briefly, the cauda epididymis was finely minced and incubated in Ham’s F-10 medium supplemented with bovine serum albumin and HEPES (Capricorn Scientific GmbH, Ebsdorfergrund, Germany) to allow spermatozoa to disperse into the medium. Sperm concentration and motility were assessed using the SCA^®^ system with a Leja counting chamber (Microptic S.L.U., Barcelona, Spain), and sperm morphology was evaluated on SpermBlue^®^-stained smears (SpermBlue^®^, Microptic S.L.U., Barcelona, Spain) under phase-contrast microscopy with a 100× oil immersion objective. For each analysis, at least 200 spermatozoa were evaluated per animal.

### 2.4. Biochemical and Hormonal Analyses

#### 2.4.1. Hormonal Biomarkers

Plasma levels of FSH and LH, and intratesticular testosterone concentrations, were measured using commercially available ELISA kits (ELK Biotechnology, Wuhan, China) according to the manufacturers’ instructions. All samples were analyzed in duplicate, and hormone concentrations were calculated from the standard curves provided with the kits.

#### 2.4.2. Oxidative Stress Biomarkers

Levels of reduced GSH and MDA were determined in testicular tissue homogenates using commercially available assay kits (ELK Biotechnology, Wuhan, China). Following centrifugation, the supernatants were analyzed. Biomarker concentrations were expressed relative to tissue weight in accordance with the manufacturer’s guidelines.

#### 2.4.3. Inflammatory Biomarkers

Testicular concentrations of TNF-α and IL-6 were quantified using commercial ELISA kits (ELK Biotechnology, Wuhan, China) according to the manufacturers’ protocols. All samples were analyzed in duplicate, and cytokine concentrations were calculated from their respective standard curves.

### 2.5. Gut Microbiota Analysis

At the end of the 10-week experimental period, fresh fecal pellets were collected from each rat prior to sacrifice for gut microbiota analysis. Fecal samples were collected by placing each animal individually in a sterile metabolic cage until spontaneous defecation occurred. Immediately after excretion, fecal pellets were aseptically transferred into sterile, DNA-free cryovials using autoclaved forceps, snap-frozen in liquid nitrogen, and stored at −80 °C until DNA extraction. To minimize diurnal and environmental variability in microbial composition, all samples were collected within a standardized 20 min time window. Microbial genomic DNA was extracted from homogenized fecal samples using the DiaRex^®^ Stool Genomic DNA Extraction Kit (DiaGen Biotechnology, Ankara, Turkey) following the manufacturer’s protocol. To ensure data quality and minimize potential contamination, extraction blanks were included and processed in parallel with all samples throughout the DNA extraction workflow. The concentration and purity of isolated DNA were assessed using a Qubit™ 4 Fluorometer in conjunction with the Qubit™ dsDNA HS Assay Kit (Thermo Fisher Scientific, Waltham, MA, USA). Samples meeting quality and concentration requirements were stored at −20 °C until downstream PCR amplification [42].

#### 16S rRNA Gene Amplification and Library Preparation

V3–V4 hypervariable regions of the bacterial 16S rRNA gene were amplified using universal primers 341F (5′-CCTACGGGNGGCWGCAG-3′) and 806R (5′-GACTACHVGGGTATCTAATCC-3′), in accordance with the Illumina 16S Metagenomic Sequencing Library Preparation Guide. PCR amplification was performed using the Nextera^®^ XT DNA Library Prep Kit (Illumina, Inc., San Diego, CA, USA) under the manufacturer’s recommended conditions. Unique dual indices were attached to each amplicon using the Nextera XT Index Kit to enable sample multiplexing. The amplified products were purified using Ampure XP Beads (Beckman Coulter, San Jose, CA, USA), quantified, and normalized before pooling to equimolar concentrations. Sequencing was conducted on an Illumina MiSeq platform using a 2 × 250 bp paired-end run with the MiSeq Reagent Kit v3 (Illumina, Inc., San Diego, CA, USA) (600-cycle) according to the manufacturer’s instructions, with all pooled libraries sequenced together in a single run to eliminate the possibility of inter-run batch effects. In addition to extraction blanks, PCR no-template controls (NTCs) were included alongside each amplification batch to monitor for potential reagent contamination or cross-contamination during library preparation.

### 2.6. Statistical Analysis

#### 2.6.1. Statistical Analysis for Sperm, Biochemical, and Hormonal Data

Data obtained from sperm parameters, oxidative stress biomarkers, and hormonal assays were analyzed using IBM SPSS Statistics, version 23.0 (IBM Corp., Armonk, NY, USA). Results are presented as the mean ± standard error of the mean (SEM). Data normality was assessed using the Shapiro–Wilk test. For normally distributed variables, one-way analysis of variance (ANOVA) was applied to compare differences among experimental groups, followed by the least significant difference (LSD) post hoc test for pairwise comparisons. Non-normally distributed data were analyzed using the Kruskal–Wallis test. A *p*-value < 0.05 was considered statistically significant.

#### 2.6.2. Bioinformatics and Statistical Analysis for Microbiome Data

Paired-end Illumina sequencing reads (2 × 250) were imported into Quantitative Insights into Microbial Ecology 2 (QIIME 2, version 2024.10). Quality filtering, trimming, denoising, and chimera removal were performed using the DADA2 plugin (version 2024.10.0) implemented in QIIME 2 to generate high-quality amplicon sequence variants (ASVs). Multiple sequence alignment and phylogenetic tree construction were performed using the MAFFT algorithm. Taxonomic classification was conducted against the SILVA 138 reference database (https://www.arb-silva.de/documentation/release-138/, accessed on 14 October 2024). Mitochondrial and chloroplast sequences were filtered out prior to downstream analysis [43].

Alpha diversity, including Observed features, Shannon index, and Chao1 richness, was calculated to assess within-sample diversity. Group-wise comparisons of alpha diversity indices were performed using the Kruskal–Wallis test with Benjamini–Hochberg false discovery rate (FDR) correction. Beta diversity was assessed using Bray–Curtis, weighted UniFrac, and unweighted UniFrac distance metrics and visualized via principal coordinate analysis (PCoA). Differences in microbial community structure among groups were evaluated using permutational multivariate analysis of variance (PERMANOVA). Differentially abundant taxa among experimental groups were identified using LEfSe with an LDA score threshold >3.0 and *p* < 0.05, as well as DESeq2 analysis with FDR correction (adjusted *p* < 0.01) [44,45]. Microbiome-related statistical analyses and visualizations were conducted using the phyloseq, vegan, and ggplot2 packages in R software (version 4.2.2).

## 3. Results

### 3.1. Evaluation of Body Weights and Relative Organ Weights

Body weight gain over the 10-week experimental period was significantly greater in the HFD group compared with the ND group (*p* < 0.05). The percentage increase in body weight was 43.59% in the ND group and 62.02% in the HFD group, confirming the successful induction of obesity in HFD-fed rats. In both P-HFD and HFD-P groups, body weight gain remained significantly higher than that observed in the ND group but was significantly lower than in the HFD group (*p* < 0.05). The percentage increases in body weight were 50.36% in the P-HFD group and 49.63% in the HFD-P group, indicating a partial attenuation of HFD-induced weight gain following prebiotic–probiotic supplementation (Table 1).

Consistent with HFD-induced metabolic alterations, relative testicular and epididymal weights were significantly reduced in the HFD group compared with the ND group (*p* < 0.05). Although both the P-HFD and HFD-P groups exhibited slightly higher relative organ weights compared with the HFD group, these differences did not reach statistical significance (*p* > 0.05). These findings indicate that prebiotic–probiotic supplementation did not reverse HFD-induced reductions in relative testicular or epididymal weights under the conditions of this study (Table 1).

### 3.2. Evaluation of Sperm Parameters

As shown in Table 2, sperm concentration was significantly reduced. Similarly, the percentage of abnormal sperm morphology was significantly increased in all HFD-fed groups compared with the ND group (*p* < 0.05). These alterations were observed not only in rats fed the HFD alone but also in those receiving concurrent or delayed prebiotic–probiotic supplementation (P-HFD and HFD-P), indicating that supplementation did not prevent HFD-induced impairments in sperm concentration or morphology under the conditions of this study. In contrast, sperm motility values, although numerically lower in HFD-fed groups, did not differ significantly among the experimental groups (*p* > 0.05).

### 3.3. Histological Examination of Testicular Tissue

**Figure 1:**
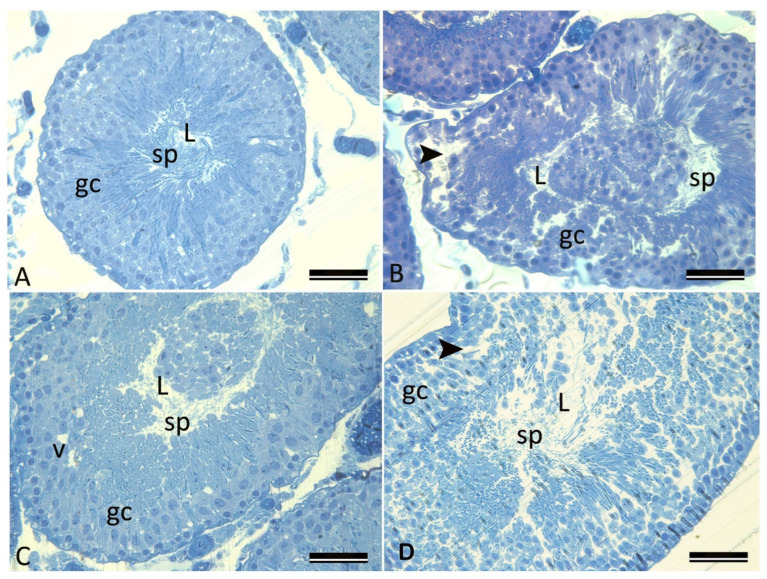
Representative H&E-stained histological sections of rat testicular tissue from the experimental groups. (**A**) ND group: Seminiferous tubules exhibit normal architecture with well-organized germinal epithelial cells (gc), abundant spermatozoa (sp) in the lumen (L), and intact basement membranes. (**B**) HFD group: Degeneration of the seminiferous tubules with disorganized germinal epithelium (gc), cytoplasmic vacuolization, widened intercellular spaces (arrowhead), and reduced spermatozoa (sp) in the lumen (L). (**C**) P-HFD group: Mild structural irregularities and vacuolation (v) in germinal epithelial cells (gc), with a moderate decrease in luminal spermatozoa (sp) within the lumen (L). (**D**) HFD-P group: Marked epithelial disorganization (gc) and degeneration with disrupted germinal cell membranes (arrowhead), and reduced spermatozoa (sp) within the lumen (L). Low magnification (×100), scale bar = 50 µm.

In the ND group, seminiferous tubules exhibited normal histoarchitecture, characterized by a well-organized germinal epithelium and intact interstitial tissue, with lumina containing abundant mature spermatozoa (Figure 1A). In contrast, the HFD group showed marked seminiferous tubular degeneration, including disruption of epithelial stratification, cytoplasmic vacuolization within germinal cells, widening intercellular spaces, and depletion of spermatids/spermatozoa from the tubular lumen. Necrotic germ cells were frequently observed, and discontinuity in the basement membrane was evident in several tubules (Figure 1B). In the P-HFD group, seminiferous tubules largely preserved their structural integrity, displaying only mild epithelial irregularities, occasional peripheral vacuolization, and partial loss of epithelial continuity. Although luminal sperm content was moderately reduced compared with the ND group, it remained noticeably higher than that observed in the HFD group (Figure 1C). Conversely, the HFD-P group exhibited severe histopathological deterioration, characterized by extensive germinal epithelial degeneration, epithelial atrophy, fragmentation of tubular walls, and near-complete disorganization of seminiferous architecture. These alterations were accompanied by a marked reduction in luminal spermatozoa (Figure 1D).

**Figure 2:**
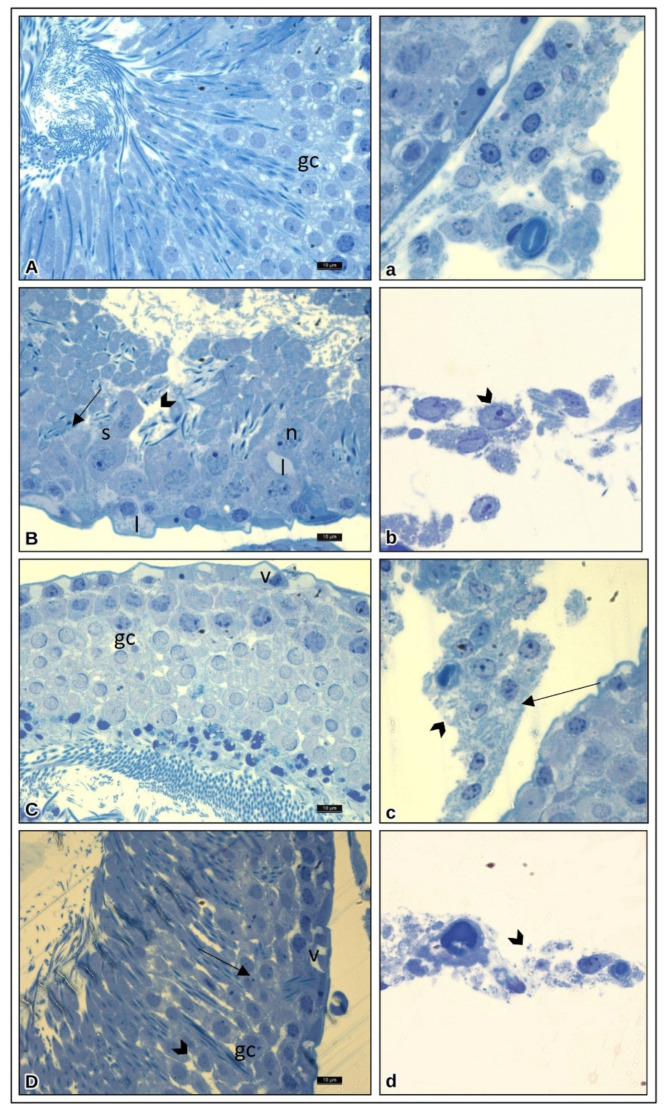
Representative high-magnification H&E-stained images illustrating histological alterations in germinal epithelium and interstitial (Leydig) cells of rat testes from the experimental groups. (**A**) ND group: Normal arrangement of germinal epithelial cells (gc) and intact seminiferous tubule structure. (**a**) Leydig cells exhibit a typical polyhedral morphology, with a centrally located nucleus and clear cytoplasmic boundaries. (**B**) HFD group: Irregular germinal epithelium (gc), presence of lipid droplets (l), swollen seminiferous tubules (s), and necrotic germ cells (n), with intercellular edema and structural disorganization (arrow) and degenerating germ cells (arrowhead). (**b**) Leydig cells exhibit shrinkage, cytoplasmic vacuolization, and membrane damage (arrowhead). (**C**) P-HFD group: Largely preserved tubular organization with mild vacuolation (v) in the outer germinal layer (gc). (**c**) Leydig cells display near-normal morphology with minor membrane irregularities (arrow) and focal cytoplasmic degeneration (arrowhead), accompanied by occasional lipid droplets. (**D**) HFD-P group: Severe epithelial degeneration (gc), fragmentation, and deformation of seminiferous tubules with extensive vacuolation (V), with disrupted seminiferous tubule architecture (arrow) and epithelial degeneration with cellular breakdown (arrowhead). (**d**) Marked Leydig cell lysis with loss of cellular integrity and pronounced membrane disruption (arrowhead). High magnification (×400), scale bar = 30 µm.

In the ND group, the germinal epithelium of the seminiferous tubules was intact and well organized, and Leydig cells exhibited typical polyhedral morphology with a centrally located nucleus (Figure 2A,a). In the HFD group, pronounced degenerative alterations were observed, including cytoplasmic lipid accumulation, cell shrinkage, membrane rupture, and focal necrosis (Figure 2B,b). In the P-HFD group, Leydig cells largely preserved near-normal morphology, displaying only minor membrane irregularities and occasional lipid droplets (Figure 2C,c). In contrast, the HFD-P group showed extensive Leydig cell lysis, diffuse cytoplasmic disintegration, and widespread membrane disruption, consistent with the severe tubular damage observed in this group (Figure 2D,d).

### 3.4. Evaluation of Serum Reproductive Hormone Levels

As shown in Table 3, serum FSH levels did not differ significantly among the experimental groups (*p* > 0.05). In contrast, serum LH levels were consistently and significantly reduced in all HFD-fed groups (HFD, P-HFD, and HFD-P), compared with the ND group (*p* < 0.05), indicating suppression of pituitary gonadotropin secretion under HFD conditions. Testicular testosterone levels were significantly elevated in the HFD and HFD-P groups compared with the ND group (*p* < 0.05). In contrast, testosterone levels in the P-HFD group did not differ significantly from those of the ND group. No significant differences in FSH, LH, or testosterone concentrations were observed among the HFD-based groups, suggesting that prebiotic–probiotic supplementation did not substantially restore systemic reproductive hormone profiles under the conditions of this study (*p* > 0.05).

### 3.5. Evaluation of Testicular Oxidative Stress Biomarkers

As presented in Table 4, no statistically significant differences were observed in testicular GSH or MDA levels among the experimental groups (*p* > 0.05). Although GSH levels decreased modestly in the HFD group compared with the ND group, this difference did not reach statistical significance. Similarly, MDA concentrations remained comparable across all groups, indicating no detectable increase in lipid peroxidation. Overall, these results demonstrate that neither HFD feeding nor prebiotic–probiotic supplementation significantly altered testicular oxidative stress markers under the experimental conditions of this study.

### 3.6. Evaluation of Testicular Inflammatory Cytokine Levels

As shown in Table 5, testicular TNF-α concentrations were significantly elevated in the HFD group compared with the ND group (*p* < 0.05). In the concurrent prebiotic–probiotic supplementation (P-HFD) group, TNF-α concentrations were significantly reduced relative to the HFD group (*p* < 0.05), indicating attenuation of HFD-induced testicular inflammation. In contrast, the HFD-P group exhibited the highest TNF-α levels among all experimental groups. TNF-α concentrations in this group were significantly higher than those observed in both the ND and P-HFD groups (*p* < 0.05), reflecting a pronounced inflammatory response when prebiotic–probiotic supplementation was initiated after prolonged HFD exposure.

Although testicular IL-6 levels increased numerically in all HFD-fed groups compared with the ND group, these differences did not reach statistical significance (*p* > 0.05). Both the P-HFD and HFD-P groups exhibited slightly lower IL-6 concentrations than the HFD group; however, these reductions were not statistically significant. Overall, these results indicate that testicular IL-6 levels were less responsive to dietary intervention or prebiotic–probiotic supplementation under the experimental conditions of this study.

### 3.7. Evaluation of Gut Microbiome

#### 3.7.1. Sequencing Statistics and Amplicon Sequence Variants (ASVs)

High-throughput sequencing generated a median of 78,258 reads per sample (range: 19,668–118,922). Following quality filtering, denoising, and chimera removal, a total of 2314 high-quality ASVs were identified using the DADA2 pipeline [46]. Raw forward and reverse read counts for each sample are provided in Supplementary Table S1 and indicate consistent sequencing depth across samples prior to denoising.

**Figure 3:**
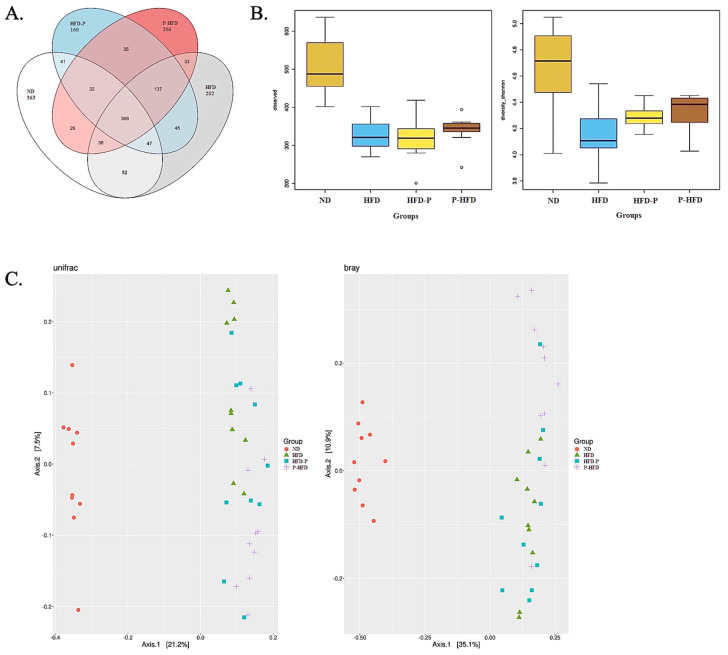
Gut microbiome diversity and community structure across experimental groups. (**A**) A Venn diagram illustrating the distribution of shared and unique genera among the ND, HFD, P-HFD, and HFD-P groups. The numbers indicate the number of genera in each unique or shared region, and the colored areas represent the corresponding experimental groups. ND rats had the highest number of unique genera, consistent with a diverse and stable microbial community. In contrast, HFD feeding substantially reduced both unique and shared genera, indicating a marked diet-induced depletion of the microbial community. (**B**) Alpha diversity metrics showing observed ASVs (left) and Shannon diversity index (right). Open circles indicate outliers. HFD markedly reduced richness and diversity compared with ND (observed ASVs: *p* = 0.00018; Shannon: *p* = 0.0032). Neither P-HFD nor HFD-P supplementation significantly improved alpha diversity relative to HFD (P-HFD: *p* = 0.4056 and 0.0821; HFD-P: *p* = 0.7336 and 0.0963 for observed ASVs and Shannon, respectively), although both groups exhibited modest upward numerical shifts. (**C**) Principal coordinates analysis (PCoA) plots based on unweighted UniFrac (left) and Bray–Curtis dissimilarity (right) metrics. Both analyses demonstrated significant between-group separation (*p* = 0.001 for each), indicating distinct microbial community structures across treatments. Each point (small circle/shape) represents one individual sample. ND: red circles; HFD: green triangles; HFD-P: teal squares; P-HFD: purple crosses.

A Venn diagram illustrating the distribution of shared and unique genera among the ND, HFD, P-HFD, and HFD-P groups is shown in Figure 3A. The ND group exhibited the highest number of unique genera. In contrast, HFD-fed rats showed a marked reduction in both unique and shared genera. Prebiotic–probiotic supplementation was associated with a numerical increase in genus-level richness in both the P-HFD and HFD-P groups; however, neither supplementation strategy restored genus-level diversity to levels comparable to those observed in the ND group, indicating incomplete restoration of overall microbial diversity.

#### 3.7.2. Microbial Diversity Across Groups

Alpha diversity was evaluated using observed ASVs and the Shannon diversity index to assess microbial richness and community evenness. The ND group exhibited significantly higher alpha diversity than the HFD group (observed ASVs: *p* = 0.00018; Shannon index: *p* = 0.0032), confirming a marked reduction in microbial diversity associated with diet-induced obesity.

In the P-HFD and HFD-P groups, no statistically significant improvements in alpha diversity were observed relative to the HFD group for either observed ASVs (*p* = 0.41, *p* = 0.73) or the Shannon index (*p* = 0.08, *p* = 0.10). However, both groups demonstrated numerical increases in diversity measures. Across all experimental groups, the ND group consistently displayed the highest alpha diversity (observed ASVs: *p* = 0.00005; Shannon index: *p* = 0.002). While the prophylactic supplementation (P-HFD) group showed a more pronounced numerical increase in richness and diversity than the therapeutic supplementation (HFD-P), neither supplementation strategy fully restored alpha diversity to levels observed in the ND group (Figure 3B). These findings indicate that HFD feeding leads to a substantial loss of microbial richness, whereas prebiotic–probiotic supplementation partially mitigates this decline.

Beta diversity analyses revealed significant differences in microbial community composition between the ND and HFD groups (*p* = 0.001 for both unweighted UniFrac and Bray–Curtis distances), highlighting a substantial diet-induced divergence in microbial structure (Figure 3C; Supplementary Table S2). When all four groups were evaluated together, PCoA ordinations based on both metrics revealed distinct clustering patterns, indicating that both dietary fat content and supplementation strategies shaped overall microbial community structure.

Both the P-HFD and HFD-P groups shifted away from the HFD cluster, indicating partial restructuring of gut microbial communities following supplementation (Figure 3C). However, neither group overlapped with the ND cluster, suggesting incomplete restoration of microbial community structure. Notably, although the P-HFD group did not occupy an intermediate position along the ND–HFD axis at the overall community-structure level, it exhibited the most pronounced taxonomic restructuring among the supplemented groups (see Section 3.7.3), consistent with a distinct, intervention-specific microbial configuration rather than a direct shift toward the ND profile.

#### 3.7.3. Relative Taxonomic Abundance Among Groups

**Figure 4:**
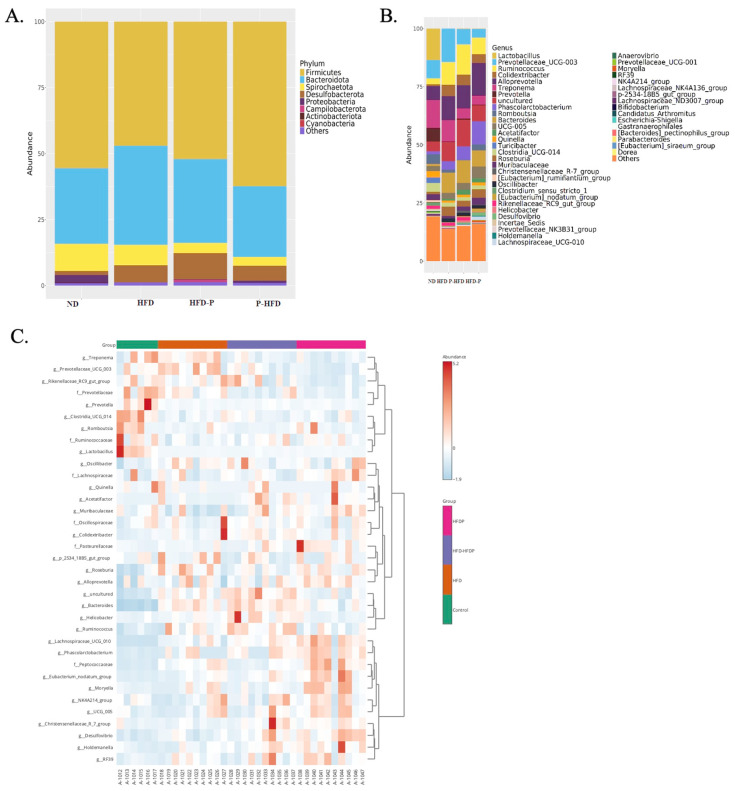
Phylum- and genus-level microbial compositions across experimental groups. (**A**) Relative abundance of dominant bacterial phyla in the ND, HFD, HFD-P, and P-HFD groups. The gut microbiota was primarily composed of *Firmicutes*, *Bacteroidota*, *Spirochaetota*, *Desulfobacterota*, and *Proteobacteria* across all groups. HFD feeding decreased the Firmicutes/Bacteroidota (F/B) ratio compared with ND, indicating diet-induced dysbiosis, whereas prophylactic supplementation (P-HFD) increased the F/B ratio, exceeding ND-like levels. (**B**) Genus-level bar plots showing the distribution of major microbial taxa across treatment groups. HFD feeding caused a marked loss of *Lactobacillus* abundance and an increase in *Prevotellaceae_UCG-003*. Both supplementation regimens partially restored *Lactobacillus* levels and reduced *Prevotellaceae_UCG-003*, with greater recovery observed in the P-HFD group. (**C**) Heatmap of the top differentially abundant genera identified across groups. HFD-fed rats displayed broad shifts in microbial composition, with increased abundance of inflammatory- or metabolically associated taxa and depletion of beneficial genera. Prebiotic–probiotic supplementation partially reversed several of these alterations, with the P-HFD group exhibiting a more ND-like clustering pattern than the HFD-P group. Each column represents one sample, and color intensity corresponds to relative abundance.

At the phylum level, Firmicutes, Bacteroidota, Spirochaetota, Desulfobacterota, and Proteobacteria were the predominant taxa across all experimental groups (Figure 4A). The Firmicutes/Bacteroidota (F/B) ratio, a commonly used indicator of gut microbial dysbiosis, was reduced in the HFD and HFD-P groups compared with the ND group. In contrast, the P-HFD group exhibited an increased F/B ratio relative to the HFD group, indicating a shift in microbial composition toward the pattern observed in ND-fed rats (Supplementary Table S3).

At the genus level (Figure 4B,C), marked compositional differences were observed among the experimental groups. Lactobacillus abundance was markedly reduced in the HFD group. Both P-HFD and HFD-P exhibited partial restoration of Lactobacillus abundance, with a more pronounced increase observed in the P-HFD group. HFD feeding was associated with a marked increase in *Prevotellaceae_UCG-003*, whereas both supplementation groups showed reduced abundance of this taxon, approaching levels observed in the ND group (Figure 4B). In contrast, Prevotella abundance was substantially reduced in all HFD-fed groups compared with the ND group, and no notable recovery was observed following supplementation. Bacteroides abundance increased 5–7-fold in HFD-fed rats and reached its highest levels in the HFD-P group. Similarly, Ruminococcus exhibited a marked increase under HFD conditions, again peaking in the HFD-P group. In contrast, Treponema abundance progressively decreased in both supplementation groups (Supplementary Table S4).

The genus Muribaculaceae, which has been associated with metabolic health, was markedly reduced by HFD feeding. Both P-HFD and HFD-P groups exhibited increased Muribaculaceae abundance compared with the HFD group (Figure 4B), with a greater numerical increase observed in the P-HFD. Despite these changes, prebiotic–probiotic supplementation did not fully re-establish a genus-level microbial profile comparable to that of the ND group.

Collectively, these findings demonstrate that HFD feeding induces a characteristic pattern of gut microbial dysbiosis, evidenced by alterations in the Firmicutes/Bacteroidota ratio, marked depletion of beneficial taxa such as Lactobacillus, Prevotella, and Muribaculaceae, and an overrepresentation of genera associated with metabolic disturbance, including Bacteroides, Ruminococcus, and *Prevotellaceae_UCG-003*. Although prebiotic–probiotic supplementation partially attenuated several HFD-induced microbial alterations, it did not fully restore the gut microbial community to the composition observed in the ND group; instead, supplementation resulted in a modest shift toward a more balanced microbial profile.

Taken together, these results indicate that the timing of microbiota-directed supplementation substantially influences its modulatory impact. While both supplementation strategies mitigated certain aspects of HFD-induced dysbiosis, the P-HFD group exhibited more pronounced taxonomic shifts than the HFD-P group, suggesting a greater microbiota-modulating effect when supplementation was initiated concurrently with HFD exposure.

#### 3.7.4. Identification of Differentially Abundant Taxa

**Figure 5:**
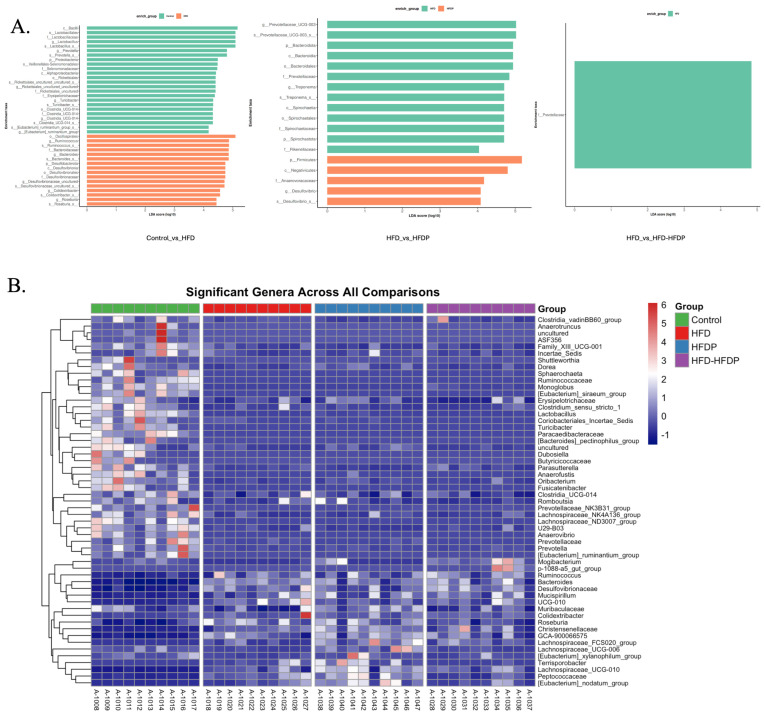
Differentially abundant taxa identified by LEfSe and DESeq2 analyses across experimental groups. (**A**) Linear discriminant analysis effect size (LEfSe) results showing genera significantly enriched between ND vs. HFD (left), HFD vs. P-HFD (middle), and HFD vs. HFD-P (right). ND rats showed higher abundances of beneficial commensals, including *Lactobacillus*, *Prevotella*, and *Eubacterium*. In contrast, HFD-fed rats showed marked enrichment of inflammation- and metabolism-associated genera, including *Ruminococcus*, *Bacteroides*, *Oscillospirales, Desulfovibrionaceae*, *Colidextribacter*, and *Roseburia*. P-HFD supplementation produced a distinct microbial profile characterized by increased *Firmicutes*, *Negativicutes*, *Anaerovoracaceae*, and *Desulfovibrio*. At the same time, LEfSe identified *Prevotellaceae* as the only taxon differing between HFD and HFD-P, indicating limited effects of therapeutic supplementation. (**B**) Heatmap illustrating DESeq2-identified differentially abundant genera across all pairwise comparisons. ND samples clustered with higher abundances of commensal, saccharolytic, and anti-inflammatory taxa, whereas HFD samples formed a separate cluster dominated by genera associated with inflammation and metabolic dysfunction. Both P-HFD and HFD-P groups exhibited only modest shifts from the HFD profile, with P-HFD showing more pronounced restructuring than HFD-P. Color intensity represents Z-score-scaled relative abundance values for each genus.

Both LEfSe and DESeq2 analyses demonstrated that HFD feeding induced robust and consistent alterations in gut microbial composition compared with the ND group (Figure 5A,B). Several taxa commonly regarded as beneficial, including *Lactobacillus*, *Prevotella*, and *Eubacterium*, were significantly enriched in the ND group. In contrast, the HFD group exhibited increased abundances of *Oscillospirales*, *Ruminococcus*, *Bacteroides*, *Desulfovibrioaceae*, *Colidextribacter*, and *Roseburia*, reflecting a pronounced diet-induced microbial shift toward genera linked to inflammatory and metabolic processes. DESeq2 analysis further corroborated these findings by identifying significant log2-fold changes in the same direction as those detected by LEfSe, thereby reinforcing the presence of a marked HFD-induced dysbiotic profile.

Comparison between the HFD and P-HFD groups revealed two clearly distinct microbial signatures (Figure 5A). The HFD group showed higher relative abundances of *Prevotellaceae_UCG-003*, Bacteroidota, *Prevotellaceae*, *Treponema*, *Spirochaetaceae*, and *Rikenellaceae*, whereas the P-HFD group showed enrichment of Firmicutes, *Negativicutes*, *Anaerovoracaceae*, and *Desulfovibrio*. These findings indicate that the P-HFD group did not re-establish the microbial community observed in the ND group but instead developed a distinct microbial structure.

LEfSe analysis comparing the HFD and HFD-P groups identified *Prevotellaceae* as the only differentially abundant taxon (LDA > 3.0, *p* < 0.05), enriched in the HFD group (Figure 5A). No taxa were significantly enriched in the HFD-P group, suggesting that prebiotic–probiotic supplementation initiated after prolonged HFD exposure had a limited impact on reversing established dysbiosis.

Consistent with DESeq2 analysis (Figure 5B; Supplementary Table S5), ND-fed animals exhibited a characteristic microbial signature enriched in commensal, saccharolytic, and anti-inflammatory taxa. In contrast, HFD samples formed a coherent cluster, dominated by inflammation-associated genera, and showed marked depletion of beneficial taxa. Both P-HFD and HFD-P groups showed only modest shifts in microbial composition. Although these shifts indicate partial microbial modulation, neither supplementation regimen restored the microbial configuration observed in the ND group. Notably, genus-level restructuring was more pronounced in the P-HFD group than in the HFD-P group, suggesting that the timing of supplementation plays a key role in shaping microbiota responses.

## 4. Discussion

The present study demonstrates that long-term HFD feeding disrupts testicular homeostasis through convergent mechanisms involving inflammation, endocrine regulation, tissue architecture, and alterations in the gut microbiota. Consistent with previous reports, our findings indicate that prolonged HFD feeding induces metabolic and inflammatory disturbances, manifested by biochemical, hormonal, and histopathological alterations in testicular tissue. Collectively, these changes indicate the development of a pronounced inflammatory–degenerative process at the testicular level. The marked elevation of TNF-α is consistent with a pro-inflammatory response to HFD feeding at the testicular level. The non-significant upward trend observed in IL-6 may be compatible with a secondary inflammatory response, although the cross-sectional, single-time-point design of this study does not allow direct assessment of the temporal sequence between TNF-α and IL-6 elevation. Taken together, these findings support the notion that HFD rapidly initiates an inflammatory cascade in the testes. Neither GSH nor MDA levels differed significantly between groups, indicating that measurable oxidative damage was not detected in testicular tissue under the conditions of this study, despite significant elevation of TNF-α. Accordingly, our original interpretation suggesting an “early, compensated” stage of oxidative stress has been revised, as such a temporal inference cannot be supported by the present cross-sectional dataset. Although oxidative stress is widely recognized as an important contributor to obesity-associated reproductive dysfunction [4,47,48,49], our findings do not support a measurable contribution of oxidative damage under the experimental conditions examined. Therefore, the contribution of oxidative stress as a mechanistic mediator between gut dysbiosis and testicular injury could not be substantiated by the GSH and MDA data obtained in the present model. Instead, the observed testicular pathology is more appropriately interpreted as predominantly inflammation-associated. Although obesity is generally characterized by reciprocal interactions between inflammation and oxidative stress [8,50,51], this interplay was not directly demonstrated in the present study. Although systemic metabolic parameters such as insulin resistance or glucose homeostasis were not directly quantified in the present study, high-fat diet feeding is well established to induce profound metabolic dysregulation, including altered lipid metabolism, adipokine imbalance, and low-grade systemic inflammation. These metabolic disturbances constitute a critical upstream axis linking dietary excess to inflammatory signaling, endocrine disruption, and testicular injury. Accordingly, the reproductive alterations observed in this study should be interpreted within an integrated gut–metabolic–testis axis, rather than as isolated testicular effects.

Spermatogenesis is tightly regulated by hormonal signals originating from the hypothalamus, pituitary, and testes, primarily through Leydig-cell-derived sex steroids and Sertoli-cell-mediated paracrine interactions [52,53]. Lifestyle factors—particularly dietary composition—play a critical role in maintaining the integrity of this axis, as inappropriate nutritional patterns are known to disrupt HPG function and impair male reproductive capacity [7,54]. Obesity-associated hormonal disturbances frequently result in a hypogonadotropic hypogonadal profile, characterized by reduced gonadotropin secretion and consequent decreases in circulating testosterone levels [54,55,56]. Excess adipose tissue contributes to this dysregulation by suppressing gonadotropin release through adipokine- and cytokine-mediated pathways involving leptin, TNF-α, and IL-6 [7,57]. Moreover, increased aromatase activity associated with obesity has been shown to enhance the conversion of testosterone to estradiol, thereby further exacerbating hormonal imbalance [58,59]. In our study, the reduction in serum LH levels accompanied by stable FSH concentrations suggests that HFD feeding partially suppresses pituitary activity within the HPG axis. Importantly, testosterone levels were quantified in testicular tissue homogenates, reflecting intratesticular testosterone concentrations rather than circulating serum testosterone. Contrary to this general trend, however, our findings revealed a significant increase in intratesticular testosterone concentrations despite reduced LH levels. This apparent dissociation likely reflects the distinct regulatory mechanisms governing intratesticular versus circulating testosterone pools. Intratesticular testosterone concentrations are substantially higher than serum levels and are regulated not only by pituitary LH but also by local paracrine and autocrine signaling within the testicular microenvironment [60,61]. This paradoxical pattern may reflect local adaptive responses within the testicular microenvironment that preserve intratesticular androgen availability despite reduced gonadotropin stimulation. One possible explanation is that metabolic stress and inflammatory signaling may transiently alter local steroidogenic regulation, thereby contributing to the maintenance of intratesticular testosterone despite reduced serum LH. Inflammatory cytokines such as TNF-α and IL-6 are known to modulate the expression of key steroidogenic genes (e.g., StAR, 3β-HSD) in Leydig cells, exerting either stimulatory or inhibitory effects depending on the magnitude and duration of exposure [62,63,64,65]. Accordingly, the elevated intratesticular testosterone levels observed in this study are compatible with a transient, localized adaptive response to inflammatory stimulation rather than sustained systemic endocrine activation or classical HPG axis dysregulation. However, this interpretation remains speculative, as steroidogenic enzyme expression and Leydig cell quantification were not directly assessed in this study. Therefore, the proposed adaptive steroidogenic response should be regarded as a biologically plausible hypothesis rather than a demonstrated mechanism.

In parallel with these multifaceted biochemical alterations—including inflammatory and hormonal changes—HFD feeding was associated with significant reductions in sperm concentration and the proportion of morphologically normal spermatozoa. Numerous studies have similarly demonstrated that excess body weight and obesity adversely affect sperm quality parameters, including concentration, motility, and morphology [66,67,68,69,70]. The concomitant decreases in relative testicular and epididymal weights observed in the present study further support the hypothesis that excessive fat intake compromises testicular trophism and functional capacity. These functional impairments were corroborated by histopathological findings, which revealed disorganization of the seminiferous tubules and partial disruption of germinal epithelial integrity. Comparable testicular structural alterations, including germ cell degeneration and epithelial atrophy, have been consistently reported in rats fed a HFD [8,69,71,72]. Collectively, these findings indicate that HFD-induced metabolic stress simultaneously compromises spermatogenic efficiency and testicular architecture, underscoring the close link between systemic metabolic dysregulation and male reproductive impairment. In light of the absence of measurable oxidative damage, these findings suggest that inflammation-associated structural and endocrine disturbances are more likely than oxidative injury to underlie the observed impairment of spermatogenesis under the present experimental conditions.

A growing body of evidence indicates that the gut microbiome plays a critical regulatory role in male reproductive physiology, particularly under conditions of metabolic or toxicological stress. The gut–testis axis refers to a bidirectional regulatory network through which gut microbiota–derived signals influence testicular function primarily via metabolic, inflammatory, and endocrine pathways. Diet-induced dysbiosis can alter microbial metabolites, promote low-grade systemic inflammation, and disrupt host metabolic homeostasis, thereby indirectly impairing steroidogenesis, spermatogenesis, and testicular immune balance. These effects are thought to arise through interconnected metabolic, inflammatory, and endocrine mechanisms, ultimately influencing HPG-axis function and testicular homeostasis. HFD, endocrine-disrupting chemicals, heavy metals, and various xenobiotics have been shown to alter intestinal microbial composition, promote systemic inflammation, and impair testicular structure, ultimately leading to reduced sperm quality in rodent models [73,74,75,76,77,78]. Consistent with these mechanistic pathways, our microbiome analyses demonstrated that HFD feeding markedly reduced microbial richness and diversity and induced profound compositional shifts, including the depletion of beneficial taxa such as *Lactobacillus*, *Muribaculaceae*, and *Prevotella*, alongside expansion of inflammation- and metabolism-associated genera such as *Bacteroides*, *Ruminococcus*, and *Prevotellaceae_UCG-003*. Notably, prophylactic supplementation was associated with partial, taxon-specific shifts toward the ND profile (e.g., restoration of *Lactobacillus abundance* and the Firmicutes/Bacteroidota ratio) without fully restoring overall community structure, as reflected by beta-diversity analysis. These distinct microbial signatures mirror the dysbiotic profiles consistently reported in HFD models and provide a biologically plausible context for interpreting the reproductive impairments observed in the present study. However, because no functional analyses of microbiota-derived metabolites or intestinal barrier integrity were performed, these interpretations remain associative and should not be regarded as direct evidence of causal mediation.

Previous research similarly indicates that HFD-induced dysbiosis is characterized by loss of commensal bacteria and enrichment of genera associated with endotoxin production and metabolic disruption [79,80]. These alterations are associated with increased lipopolysaccharide (LPS) production, impairment of intestinal barrier integrity, and activation of systemic inflammatory pathways that may adversely affect testicular function [74,76,81,82]. Consistent with these outcomes, HFD-fed rodents typically exhibit seminiferous tubular degeneration, loss of germinal epithelium, reduced spermatogenic scores, and increased apoptotic activity in testicular tissue [74]. Across studies, gut dysbiosis has been associated with reductions in sperm concentration, motility, and viability, as well as increased morphological abnormalities, together with disturbances in steroidogenesis and inflammatory signaling. Comparable microbiota-associated reproductive alterations have also been reported in toxicant-exposure models, including bisphenols, phthalates, and strobilurin fungicides [75,78]. Collectively, these findings support the concept that microbiota-associated inflammatory and metabolic disturbances may contribute to male reproductive dysfunction and provide biological context for the microbiome alterations observed in the present study.

Both preventive and therapeutic administrations of the prebiotic–probiotic combination attenuated excessive body weight gain induced by HFD feeding, indicating beneficial metabolic effects of the intervention. Although complete normalization of body weight was not achieved, this attenuation suggests modulation of energy homeostasis and lipid metabolism. These findings are consistent with previous studies showing that synbiotic supplementation attenuates HFD-induced weight gain through multiple metabolic mechanisms. Several studies have shown that probiotics regulate lipid metabolism and improve systemic energy balance by promoting fatty acid oxidation while suppressing de novo lipogenesis [83,84]. In addition, probiotic strains such as *Lactiplantibacillus plantarum* have been shown to restore gut microbial composition, strengthen intestinal barrier integrity, and reduce systemic inflammation and endotoxemia [85,86]. Taken together, these findings support the hypothesis that microbiota-targeted interventions can alleviate diet-induced metabolic disturbances by modulating the gut–metabolic axis and improving systemic metabolic homeostasis. However, improvements in systemic metabolic status alone did not fully explain the reproductive outcomes observed in the present study. Despite comparable attenuation of body weight gain in both the P-HFD and HFD-P groups, their reproductive outcomes diverged markedly, with only the P-HFD group exhibiting preserved testicular architecture and partial hormonal normalization. This dissociation suggests that the timing of intervention, together with the degree of local testicular inflammation, rather than systemic adiposity per se, may be a major determinant of reproductive impairment in this model.

Protective modulation of the gut–testis axis has gained increasing research attention, as interventions targeting microbial homeostasis have been shown to benefit both gut ecology and male reproductive function. In the present study, the contribution of the gut microbiota to reproductive protection is interpreted as an associative relationship supported by integrated phenotypic findings and previous experimental evidence, rather than direct causal proof. Among these, probiotic strains belonging to *Lactiplantibacillus plantarum*—including RS20D and LP1008—have demonstrated notable efficacy in experimental models. These strains enhance microbial diversity, suppress pathogenic taxa, reduce systemic inflammatory signaling, and stabilize testicular antioxidant defenses. They have also been reported to normalize steroidogenic pathways, increase testicular testosterone concentrations, and improve sperm concentration, motility, morphology, and testicular histoarchitecture [74,77]. Mineral-based interventions such as strontium chloride similarly modulate specific microbial taxa associated with metabolic dysfunction and enhance steroidogenic gene expression, although their effects on sperm quality appear less consistent [73]. In our study, concomitant administration of the prebiotic–probiotic mixture during HFD feeding exerted pronounced structural and anti-inflammatory protective effects. Testicular TNF-α levels in the P-HFD group were restored to values that were no longer significantly different from those of the ND group, indicating substantial attenuation of the inflammatory response induced by HFD. Such anti-inflammatory activity may be associated with alleviating HFD-induced gut dysbiosis, as microbiota-directed supplementation may restore microbial balance, strengthen the intestinal barrier integrity, and limit systemic immune activation [87,88,89,90]. The resulting reduction in inflammatory burden may downregulate NF-κB-dependent signaling pathways, thereby contributing to the restoration of testicular homeostasis [21,91]. In parallel, intratesticular testosterone levels in the concurrently supplemented group returned to values comparable with the ND group despite persistently reduced serum LH. This finding may be compatible with partial recovery of local Leydig cell steroidogenic function but does not constitute direct evidence of such recovery. Importantly, this hormonal change was not accompanied by improvements in sperm concentration, motility, or morphology and should therefore not be interpreted as evidence of functional reproductive recovery. Accordingly, microbiota-related changes are interpreted as modulatory factors that may influence the inflammatory tone and testicular homeostasis, rather than directly determining reproductive outcomes. Nevertheless, definitive causal attribution of these protective effects to microbiota modulation would require targeted functional approaches, such as microbiota depletion, transplantation, or gnotobiotic models, which were beyond the scope of the present study.

An intriguing divergence emerged in the P-HFD group: concurrent supplementation preserved seminiferous epithelial integrity and Leydig cell architecture, accompanied by restoration of intratesticular testosterone to values comparable with the ND group and substantial suppression of TNF-α, yet sperm concentration and morphology remained largely unimproved. This discrepancy likely reflects several interconnected mechanisms. First, structural preservation may chronologically precede functional recovery. A complete spermatogenic cycle in rats, including epididymal transit and maturation, requires approximately 70 days [35]. Therefore, many of the spermatozoa analyzed at the end of the experiment had already entered spermatogenesis during the initial phase of HFD exposure, when testicular injury was most pronounced, and additional spermatogenic cycles may therefore be required before measurable improvements in sperm quality become evident. Second, although TNF-α was substantially reduced, residual inflammatory activity may have persisted. Even modest residual inflammatory activity may impair Sertoli cell function, blood–testis barrier integrity, and germ cell adhesion [92], thereby limiting spermatogenic recovery despite preservation of tissue architecture. Finally, persistent suppression of LH suggests incomplete recovery of the hypothalamic–pituitary–gonadal axis. Because normal spermatogenesis depends on coordinated gonadotropin signaling and adequate intratesticular androgen production [55], incomplete endocrine recovery may have constrained functional improvement despite the observed histological protection.

In contrast, supplementation initiated after obesity and testicular injury were established was markedly less effective. When administered therapeutically, the prebiotic–probiotic combination did not reduce testicular TNF-α, which instead remained significantly elevated relative to both the ND and P-HFD groups. Although TNF-α levels were numerically higher than those observed in the untreated HFD group, this difference was not statistically significant and should therefore be interpreted cautiously. This pattern was corroborated by histopathological evaluation: the HFD-P group exhibited severe epithelial degeneration, fragmentation and deformation of seminiferous tubules, extensive vacuolation, and marked Leydig cell lysis with loss of membrane integrity—alterations that were, in several respects, more pronounced than those observed in the untreated HFD group, suggesting that therapeutic intervention initiated after the establishment of obesity was considerably less effective in preserving testicular architecture. Although intratesticular testosterone was elevated in this group, this finding should not be interpreted as evidence of functional recovery. As discussed above, it may reflect altered local steroidogenic regulation; however, this interpretation remains hypothetical because steroidogenic enzyme expression was not evaluated. Consistent with this interpretation, neither sperm concentration nor morphology demonstrated meaningful improvement, with both parameters remaining comparable to those of the untreated HFD group. Together, these findings indicate that delayed supplementation was insufficient to restore either testicular structure or functional reproductive outcomes under the experimental conditions of this study.

Microbiome profiling confirmed that neither supplementation regimen restored the microbial community to its baseline configuration. Instead, both interventions generated compositionally distinct alternative community states, consistent with ecological models suggesting that microbiota-directed therapies reshape dysbiotic communities toward new equilibria rather than restoring their original composition [93]. Although several taxa enriched in the P-HFD group (e.g., *Negativicutes*, *Anaerovoracaceae*, *Desulfovibrio*) are conventionally associated with dysbiosis, emerging evidence indicates that their functional roles are highly context-dependent and may vary according to host metabolic state [94,95,96,97,98,99]. These findings suggest that early supplementation promotes the establishment of an intervention-specific microbial ecosystem, distinct from both the ND and HFD microbiota. Whether this alternative community state confers long-term functional benefits remains to be determined.

This study has several limitations. First, intermediate mechanistic biomarkers linking gut dysbiosis to testicular inflammation—such as intestinal tight junction proteins and fecal short-chain fatty acids—were not assessed. Accordingly, the proposed involvement of the gut–testis axis is supported by associative evidence rather than direct mechanistic demonstration. Second, the experimental design did not include probiotic-only or prebiotic-only comparator groups, precluding discrimination between the individual, synergistic, or potential postbiotic contributions of the intervention. Third, although the sample size (*n* = 10 per group) is consistent with those commonly used in experimental rodent reproductive toxicology studies, no formal a priori power analysis was performed. Consequently, some non-significant findings (e.g., IL-6 and certain alpha-diversity indices) may reflect limited statistical power rather than the true absence of biological effects. Fourth, histopathological evaluation was descriptive rather than based on a standardized scoring system (e.g., Johnsen score), limiting quantitative comparison across studies. Fifth, direct correlation analyses between microbial taxa and host physiological parameters (e.g., testosterone, LH, TNF-α, and sperm parameters) were not performed, limiting mechanistic interpretation of microbiota–host associations. Finally, randomization procedures were not formally implemented, investigators were not blinded during sample collection or analysis, and potential cage effects were not incorporated into microbiome analyses. These factors may have introduced observer bias and unaccounted intra-cage variability.

Overall, this study demonstrates that HFD feeding triggers a multi-level cascade of inflammatory, endocrine, and structural disturbances, accompanied by marked alterations in gut microbiota composition that collectively impair male reproductive health. Notably, the timing of intervention was a key determinant of treatment efficacy: prophylactic supplementation modulated gut microbial composition, reduced testicular inflammation, and preserved seminiferous architecture, whereas therapeutic supplementation initiated after obesity-related injury had become established conferred minimal benefit and failed to prevent progressive testicular degeneration. Importantly, even prophylactic supplementation did not improve sperm concentration or morphology, indicating that structural and anti-inflammatory protection alone is insufficient to achieve rapid functional recovery. These findings suggest that microbiota-targeted interventions may be most effective when initiated before advanced testicular damage develops. Future studies incorporating longer intervention periods, strain-specific functional characterization, and causal approaches such as microbiota depletion, fecal microbiota transplantation, or gnotobiotic models will be essential to determine whether prolonged modulation of the gut–metabolism–testis axis can ultimately facilitate recovery of spermatogenesis.

Although caution is warranted when extrapolating animal data to humans, these findings may have translational relevance for obesity-associated male reproductive dysfunction. Because obesity-related hypogonadism and impaired spermatogenesis often develop gradually and may already be established at the time of clinical presentation, microbiota-targeted strategies may have greater therapeutic potential as preventive rather than corrective interventions. Future clinical studies examining the influence of intervention timing across different stages of obesity-associated metabolic dysfunction will be important to determine whether this temporal pattern applies to human reproductive health.

## 5. Conclusions

This study demonstrates that long-term HFD feeding induces coordinated alterations in gut microbiota composition, testicular inflammation, endocrine regulation, and tissue architecture, collectively contributing to impaired male reproductive function. Prebiotic–probiotic supplementation exhibited a clear timing-dependent effect: concurrent administration attenuated testicular inflammation, preserved seminiferous architecture, and partially reshaped the gut microbiota, whereas supplementation initiated after metabolic injury conferred only marginal benefit and was associated with more pronounced inflammation and structural degeneration than HFD alone. Importantly, neither supplementation regimen improved sperm concentration or morphology, indicating that structural and anti-inflammatory protection alone was insufficient to restore spermatogenic function within the experimental timeframe. These findings suggest that microbiota-targeted interventions may be most effective when initiated before chronic testicular injury becomes established.

Overall, our findings highlight the importance of intervention timing in microbiota-directed strategies targeting the gut–metabolism–testis axis. Although further mechanistic and translational studies are required, early prebiotic–probiotic intervention may represent a more promising approach than delayed treatment for mitigating obesity-associated male reproductive dysfunction.

## Figures and Tables

**Table 1:** Body weights and relative testis and epididymis weights of the groups.

Groups	Body Weight (g)	Relative Testis Weight (g/100 g Body Weight)	Relative Epididymis Weight (g/100 g Body Weight)
ND	362.90 ± 10.63	0.38 ± 0.008	0.16 ± 0.004
HFD	443.50 ± 10.61 (*)	0.31 ± 0.008 (*)	0.13 ± 0.003 (*)
P-HFD	390.40 ± 16.73 (*, +)	0.32 ± 0.01 (*)	0.14 ± 0.007 (*)
HFD-P	384.40 ± 6.70 (*, +)	0.33 ± 0.006 (*)	0.14 ± 0.004 (*)

ND: Rats were fed a standard control diet for 10 weeks. HFD: Rats were fed a high-fat diet for 10 weeks. P-HFD: Rats were fed a high-fat diet and simultaneously administered a prebiotic–probiotic combination (1.03 × 10^9^ CFU/kg/day of freeze-dried bacterial strains and 31 mg/kg/day of inulin) via oral gavage for 10 weeks. HFD-P: Rats were fed a high-fat diet for the first five weeks, followed by an additional five weeks of high-fat feeding combined with daily administration of the prebiotic–probiotic combination at the same dose described above. Data are presented as mean ± SEM. Statistical analysis was performed using one-way ANOVA followed by the LSD post hoc test for normally distributed data and the Kruskal–Wallis test for non-normally distributed variables. (*) Significantly different from ND (*p* < 0.05). (+) Significantly different from HFD (*p* < 0.05).

**Table 2:** Sperm parameters of the groups.

Groups	Sperm Concentration (10^6^/mL)	Sperm Motility (%)	Abnormal Sperm Morphology (%)
ND	3.27 ± 0.28	90.43 ± 2.00	19.69 ± 1.30
HFD	1.97 ± 0.15 (*)	80.74 ± 4.21	33.06 ± 2.40 (*)
P-HFD	1.99 ± 0.32 (*)	87.87 ± 2.60	34.31 ± 2.58 (*)
HFD-P	2.17 ± 0.41 (*)	85.37 ± 2.66	35.44 ± 2.21 (*)

ND: Rats were fed a standard control diet for 10 weeks. HFD: Rats were fed a high-fat diet for 10 weeks. P-HFD: Rats were fed a high-fat diet and simultaneously administered a prebiotic–probiotic combination (1.03 × 10^9^ CFU/kg/day of freeze-dried bacterial strains and 31 mg/kg/day of inulin) via oral gavage for 10 weeks. HFD-P: Rats were fed a high-fat diet for the first five weeks, followed by an additional five weeks of high-fat feeding combined with daily administration of the prebiotic–probiotic combination at the same dose described above. Data are presented as mean ± SEM. Statistical analysis was performed using one-way ANOVA followed by the LSD post hoc test for normally distributed variables. (*) Significantly different from ND (*p* < 0.05).

**Table 3:** Serum FSH and LH levels and intratesticular testosterone concentrations of the groups.

Groups	FSH (ng/mL)	LH (mIU/mL)	Testosterone (ng/mL)
ND	0.51 ± 0.08	1.32 ± 0.20	7.4 ± 0.67
HFD	0.47 ± 0.05	0.55 ± 0.12 (*)	9.97 ± 0.67 (*)
P-HFD	0.45 ± 0.07	0.64 ± 0.09 (*)	9.11 ± 0.33
HFD-P	0.47 ± 0.07	0.61 ± 0.09 (*)	10.32 ± 0.67 (*)

ND: Rats were fed a standard control diet for 10 weeks. HFD: Rats were fed a high-fat diet for 10 weeks. P-HFD: Rats were fed a high-fat diet and simultaneously administered a prebiotic–probiotic combination (1.03 × 10^9^ CFU/kg/day of freeze-dried bacterial strains and 31 mg/kg/day of inulin) via oral gavage for 10 weeks. HFD-P: Rats were fed a high-fat diet for the first five weeks, followed by an additional five weeks of high-fat feeding combined with daily administration of the prebiotic–probiotic combination at the same dose described above. Data are presented as mean ± SEM. Statistical analysis was performed using one-way ANOVA followed by the LSD post hoc test for normally distributed variables. (*) Significantly different from ND (*p* < 0.05).

**Table 4:** Testicular GSH and MDA levels of the groups.

Groups	GSH (µM)	MDA (mcg/mL)
ND	27.03 ± 2.37	20.79 ± 0.86
HFD	24.14 ± 1.13	19.73 ± 0.56
P-HFD	26.97 ± 1.20	21.50 ± 0.55
HFD-P	25.40 ± 1.18	20.30 ± 0.46

ND: Rats were fed a standard control diet for 10 weeks. HFD: Rats were fed a high-fat diet for 10 weeks. P-HFD: Rats were fed a high-fat diet and simultaneously administered a prebiotic–probiotic combination (1.03 × 10^9^ CFU/kg/day of freeze-dried bacterial strains and 31 mg/kg/day of inulin) via oral gavage for 10 weeks. HFD-P: Rats were fed a high-fat diet for the first five weeks, followed by an additional five weeks of high-fat feeding combined with daily administration of the prebiotic–probiotic combination at the same dose described above. Data are presented as mean ± SEM. Statistical analysis was performed using one-way ANOVA followed by the LSD post hoc test for normally distributed variables. No statistically significant differences among groups were detected for GSH or MDA (*p* > 0.05); accordingly, no symbols appear in this table.

**Table 5:** Testicular TNF-α and IL-6 levels of the groups.

Groups	TNF-α (pg/mL)	IL-6 (pg/mL)
ND	37.85 ± 2.31	13.13 ± 0.99
HFD	45.52 ± 2.53 (*)	16.9 ± 1.39
P-HFD	39 ± 1.64 (+)	14.01 ± 1.07
HFD-P	52.24 ± 3.63 (*, !)	15.36 ± 1.15

ND: Rats were fed a standard control diet for 10 weeks. HFD: Rats were fed a high-fat diet for 10 weeks. P-HFD: Rats were fed a high-fat diet and simultaneously administered a prebiotic–probiotic combination (1.03 × 10^9^ CFU/kg/day of freeze-dried bacterial strains and 31 mg/kg/day of inulin) via oral gavage for 10 weeks. HFD-P: Rats were fed a high-fat diet for the first five weeks, followed by an additional five weeks of high-fat feeding combined with daily administration of the prebiotic–probiotic combination at the same dose described above. Abbreviations: TNF-α, tumor necrosis factor alpha; IL-6, interleukin-6. Data are presented as mean ± SEM. Statistical analysis was performed using one-way ANOVA followed by the LSD post hoc test for normally distributed variables. (*) Significantly different from ND (*p* < 0.05). (+) Significantly different from HFD (*p* < 0.05). (!) Significantly different from P-HFD (*p* < 0.05).

## Data Availability

The original contributions presented in this study are included in the article/Supplementary Materials. Further inquiries can be directed to the corresponding author.

## Supplementary Materials

The following supporting information can be downloaded at: https://www.mdpi.com/article/10.3390/microorganisms14071566/s1, Table S1. Sequencing read counts for forward and reverse reads across all samples; Table S2. PERMANOVA results comparing beta-diversity distances among experimental groups; Table S3. Relative abundance of major phyla across groups (%); Table S4. Relative abundance of major genera across groups (%); Table S5. Differentially abundant genera identified by DESeq2 across groups.
