# Supplementary material for: Timing-Dependent Effects of Prebiotic–Probiotic Supplementation on High-Fat-Diet-Induced Testicular Dysfunction and Gut Microbiota Alterations in Rats
Source: Microorganisms. 2026 Jul 17;14(7):1566. doi: 10.3390/microorganisms14071566 (PMC13413739; doi:10.3390/microorganisms14071566)
Supplement: Supplementary file 1 [file microorganisms-14-01566-s001.zip › microorganisms-4355049-supplementary.pdf]

Supplementary Table S1. Sequencing read counts for forward and reverse reads across all samples.

Sample No	Forward sequence count	Reverse sequence count
A-1027	118922	118922
A-1035	115554	115554
A-1041	109406	109406
A-1047	102879	102879
A-1038	98004	98004
A-1039	97752	97752
A-1043	92271	92271
A-1010	88897	88897
A-1019	84330	84330
A-1018	83160	83160
A-1026	81998	81998
A-1008	81593	81593
A-1034	81458	81458
A-1042	81377	81377
A-1017	80898	80898
A-1045	80432	80432
A-1031	80017	80017
A-1020	79383	79383
A-1025	79373	79373
A-1015	79295	79295
A-1016	77221	77221
A-1022	77166	77166
A-1029	74409	74409
A-1023	72526	72526
A-1028	71411	71411
A-1044	70734	70734
A-1011	70414	70414
A-1021	70310	70310
A-1046	68074	68074
A-1036	67587	67587
A-1014	65150	65150
A-1009	63274	63274
A-1013	60419	60419
A-1024	54905	54905
A-1030	54455	54455
A-1033	53194	53194
A-1012	50165	50165
A-1040	40744	40744

A-1032	27758	27758
A-1037	19668	19668

Forward and reverse sequence counts generated after demultiplexing and before quality filtering. Balanced read counts across paired-end reads indicate consistent sequencing performance and absence of technical bias.

Supplementary Table S2. PERMANOVA results comparing beta-diversity distances among experimental groups.

Df	SumOfSqs	R2	F	Pr(>F)	Comparison
Bray-Curtis	0.292894856	0.103512288	2.078356644	0.009	HFD vs HFD-P
WeightedUnifrac	0.004350221	0.122033767	2.50192743	0.019	HFD vs HFD-P
UnweightedUnifrac	0.316790769	0.097176736	1.937457004	0.002	HFD vs HFD-P
Bray-Curtis	0.467729777	0.170943767	3.711434386	0.002	HFD vs P-HFD
WeightedUnifrac	0.009068874	0.245326614	5.851377726	0.002	HFD vs P-HFD
UnweightedUnifrac	0.358730393	0.115589602	2.352542253	0.001	HFD vs P-HFD
Bray-Curtis	2.100773831	0.431908457	13.68503426	0.001	ND vs HFD
WeightedUnifrac	0.028164542	0.394927332	11.74849297	0.001	ND vs HFD
UnweightedUnifrac	1.132951208	0.291075775	7.390583873	0.001	ND vs HFD

Pairwise and overall PERMANOVA analyses based on Bray–Curtis, unweighted UniFrac, and weighted UniFrac distance matrices are presented. The table reports pseudo-F statistics, R^2 values, and p-values, indicating the significance of group-level differences in gut microbial community structure across ND, HFD, P-HFD, and HFD-P groups.

ND: Rats fed with a normal diet for 10 weeks. HFD: Rats fed with a high-fat diet for 10 weeks. P-HFD: Rats receiving a high-fat diet along with a simultaneous gastric gavage administration of 1.03×10^9 CFU/kg/day of freeze-dried bacterial mixture and 31 mg/kg/day of inulin for 10 weeks. HFD-P: Rats fed with a high-fat diet for 5 weeks, followed by an additional 5 weeks of high-fat diet along with a simultaneous gastric gavage administration of 1.03×10^9 CFU/kg/day of freeze-dried bacterial mixture and 31 mg/kg/day of inulin.

Supplementary Table S3. Relative abundance of major phyla across groups (%).

**Phylium**	**ND (%)**	**HFD (%)**	**P-HFD (%)**	**HFD-P (%)**
Firmicutes	53,834	48,758	61,488	51,634
Bacteroidota	29,83	36,144	27,326	32,499
Firmicutes / Bacteroidota	**1,804**	**1,35**	**2,250**	**1,589**
Spirochaetota	10,89	7,327	3,544	4,071,
Desulfobacterota	1,962	6,647	5,792	9.343
Proteobacteria	2,816	0,366	1,224	0,644
Campilobacterota	0,221	0,387	0,249	1,054
Actinobacteriota	0,262	0,034	0,15	0,085
Cyanobacteria	0,104	0,162	0,052	0,244

Relative phylum-level abundances and Firmicutes/Bacteroidota (F/B) ratios in the ND, HFD, P-HFD, and HFD-P groups. Values are presented as percentage means. The F/B ratio was calculated from the proportional abundances of the two phyla.

ND: Rats fed with a normal diet for 10 weeks. HFD: Rats fed with a high-fat diet for 10 weeks. P-HFD: Rats receiving a high-fat diet along with a simultaneous gastric gavage administration of 1.03×10^9 CFU/kg/day of freeze-dried bacterial mixture and 31 mg/kg/day of inulin for 10 weeks. HFD-P: Rats fed with a high-fat diet for 5 weeks, followed by an additional 5 weeks of high-fat diet along with a simultaneous gastric gavage administration of 1.03×10^9 CFU/kg/day of freeze-dried bacterial mixture and 31 mg/kg/day of inulin.

Supplementary Table S4. Relative abundance of major genera across groups (%).

Genus	ND (%)	HFD (%)	P-HFD (%)	HFD-P (%)
Treponema	12,63	8,745	4,065	4,673
Lactobacillus	12,106	0,187	0,535	0,335
Prevotella	6,273	0,75	0,761	0,917
Ruminococcus	2,727	9,807	7,495	12,57
Bacteroides	1,426	8,418	7,207	10,177

Relative abundances (%) of selected genera in the ND, HFD, P-HFD, and HFD-P groups. Values represent mean percentages obtained from 16S rRNA sequencing–based taxonomic profiling.

ND: Rats fed with a normal diet for 10 weeks. HFD: Rats fed with a high-fat diet for 10 weeks. P-HFD: Rats receiving a high-fat diet along with a simultaneous gastric gavage administration of 1.03×10^9 CFU/kg/day of freeze-dried bacterial mixture and 31 mg/kg/day of inulin for 10 weeks. HFD-P: Rats fed with a high-fat diet for 5 weeks, followed by an additional 5 weeks of high-fat diet along with a simultaneous gastric gavage administration of 1.03×10^9 CFU/kg/day of freeze-dried bacterial mixture and 31 mg/kg/day of inulin.

Supplementary Table S5. Differentially abundant genera identified by DESeq2 across groups.

Comparison	baseMean	log2FoldChange	lfcSE	stat	Pvalue	padj	Genus
HFD vs ND	2750.03	-7.14	0.63	-11.34	8.66E-30	1.28E-27	Lactobacillus
HFD vs ND	109.47	-11.62	1.03	-11.25	2.41E-29	1.78E-27	Anaerovibrio
HFD vs ND	65.30	-10.87	1.06	-10.27	9.99E-25	4.93E-23	[Eubacterium]_siraeum_group
HFD vs ND	116.12	-11.70	1.23	-9.55	1.28E-21	4.31E-20	Lachnospiraceae_ND3007_group
HFD vs ND	2896.43	2.25	0.26	8.78	1.59E-18	3.92E-17	Bacteroides
HFD vs ND	1060.17	-3.39	0.41	-8.17	2.96E-16	6.06E-15	Prevotella
HFD vs ND	499.51	-9.19	1.13	-8.16	3.28E-16	6.06E-15	Turicibacter
HFD vs ND	72.79	-8.45	1.13	-7.50	6.41E-14	1.05E-12	Monoglobus
HFD vs ND	43.06	-10.27	1.41	-7.28	3.36E-13	4.51E-12	Ruminococcaceae
HFD vs ND	99.90	-9.97	1.50	-6.63	3.45E-11	3.93E-10	Prevotellaceae_NK3B31_group
HFD vs ND	27.45	-3.98	0.60	-6.60	4.10E-11	4.34E-10	Oribacterium
HFD vs ND	168.65	-9.39	1.45	-6.48	9.12E-11	9.00E-10	[Eubacterium]_ruminantium_group
HFD vs ND	46.70	-10.39	1.65	-6.29	3.25E-10	3.01E-09	[Bacteroides]_pectinophilus_group
HFD vs ND	24.40	-25.10	4.01	-6.25	4.04E-10	3.51E-09	Shuttleworthia
HFD vs ND	75.53	-9.89	1.58	-6.24	4.34E-10	3.57E-09	Fusicatenibacter
HFD vs ND	83.85	-3.77	0.62	-6.12	9.57E-10	7.45E-09	Dorea
HFD vs ND	327.92	3.81	0.62	6.10	1.04E-09	7.70E-09	Lachnospiraceae_UCG-010
HFD vs ND	12.87	-8.52	1.45	-5.86	4.61E-09	3.25E-08	Sphaerochaeta
HFD vs ND	102.79	-3.30	0.56	-5.85	4.91E-09	3.28E-08	Parasutterella
HFD vs ND	307.23	2.60	0.45	5.84	5.10E-09	3.28E-08	[Eubacterium]_nodatum_group
HFD vs ND	16.91	4.66	0.81	5.76	8.17E-09	5.04E-08	Christensenellaceae
HFD vs ND	9.45	-23.72	4.13	-5.74	9.51E-09	5.63E-08	Anaerotruncus
HFD vs ND	12.82	14.44	3.18	4.54	5.57E-06	3.17E-05	p-1088-a5_gut_group
HFD vs ND	55.63	-2.59	0.57	-4.53	5.85E-06	3.21E-05	Family_XIII_UCG-001

HFD vs ND	235.45	-2.70	0.60	-4.51	6.34E-06	3.35E-05	Ruminococcaceae
HFD vs ND	222.89	1.58	0.35	4.48	7.42E-06	3.79E-05	GCA-900066575
HFD vs ND	40.03	-5.60	1.31	-4.29	1.81E-05	8.93E-05	Mogibacterium
HFD vs ND	1182.46	-1.79	0.42	-4.27	1.92E-05	9.19E-05	Clostridia_UCG-014
HFD vs ND	31.75	-4.04	0.99	-4.08	4.58E-05	2.05E-04	Lachnospiraceae_UCG-006
HFD vs ND	2735.01	1.62	0.40	4.02	5.84E-05	2.40E-04	Desulfovibrionaceae
HFD vs ND	81.92	-2.43	0.61	-3.95	7.98E-05	3.19E-04	Erysipelotrichaceae
HFD vs ND	3551.17	1.46	0.38	3.85	1.20E-04	4.66E-04	Ruminococcus
HFD vs ND	15.76	5.14	1.37	3.74	1.81E-04	6.53E-04	Lachnospiraceae_FCS020_group
HFD vs ND	173.65	-3.45	0.94	-3.66	2.49E-04	8.76E-04	Lachnospiraceae_NK4A136_group
HFD vs ND	57.19	-2.84	0.83	-3.44	5.83E-04	1.96E-03	[Eubacterium]_xylanophilum_group
HFD vs ND	1073.28	-1.49	0.44	-3.37	7.52E-04	2.45E-03	Muribaculaceae
HFD vs ND	1639.90	1.58	0.48	3.32	9.09E-04	2.86E-03	Colidextribacter
HFD vs ND	1276.32	-2.31	0.71	-3.27	1.07E-03	3.23E-03	Romboutsia
HFD vs ND	52.77	-10.56	3.26	-3.24	1.19E-03	3.51E-03	Butyricicoccaceae
HFD vs ND	26.64	-2.29	0.71	-3.22	1.30E-03	3.78E-03	Clostridia_vadinBB60_group
HFD vs ND	345.19	-3.35	1.05	-3.20	1.37E-03	3.91E-03	Clostridium_sensu_stricto_1
HFD vs ND	1242.29	1.28	0.40	3.17	1.52E-03	4.18E-03	Roseburia
HFD vs ND	19.01	-4.28	1.47	-2.90	3.70E-03	9.96E-03	Prevotellaceae
HFD-P vs HFD	40.03	6.02	1.31	4.61	4.05E-06	6.65E-04	Mogibacterium
P-HFD vs HFD	12.82	-24.12	3.18	-7.59	3.21E-14	5.27E-12	p-1088-a5_gut_group
P-HFD vs HFD	3731.80	-2.07	0.43	-4.84	1.30E-06	1.07E-04	Prevotellaceae_UCG-003
P-HFD vs HFD	31.75	4.23	0.99	4.27	1.93E-05	1.05E-03	Lachnospiraceae_UCG-006
P-HFD vs HFD	40.03	5.08	1.31	3.89	1.00E-04	4.10E-03	Mogibacterium
P-HFD vs HFD	60.10	1.69	0.47	3.61	3.07E-04	8.38E-03	Anaerovoracaceae
P-HFD vs HFD	307.23	1.58	0.44	3.61	3.02E-04	8.38E-03	[Eubacterium]_nodatum_group
P-HFD vs HFD	57.19	2.92	0.82	3.54	3.98E-04	9.31E-03	[Eubacterium]_xylanophilum_group

The table summarizes genus-level differences in relative abundance between groups (ND, HFD, P-HFD, HFD-P) based on DESeq2 analysis. Columns include base mean abundance, log2 fold-change, standard error, Wald test statistic, raw p-values, adjusted p-values (Benjamini–Hochberg), and taxonomic annotation. Positive log2 fold-change values indicate genera enriched in the first-listed group of each comparison.

ND: Rats fed with a normal diet for 10 weeks. HFD: Rats fed with a high-fat diet for 10 weeks. P-HFD: Rats receiving a high-fat diet along with a simultaneous gastric gavage administration of 1.03×10^9 CFU/kg/day of freeze-dried bacterial mixture and 31 mg/kg/day of inulin for 10 weeks. HFD-P: Rats fed with a high-fat diet for 5 weeks, followed by an additional 5 weeks of high-fat diet along with a simultaneous gastric gavage administration of 1.03×10^9 CFU/kg/day of freeze-dried bacterial mixture and 31 mg/kg/day of inulin.
